# Early-life carriage and antibiotic resistance of *Streptococcus pneumoniae* in infants from Sierra Leone

**DOI:** 10.3389/fmicb.2026.1822296

**Published:** 2026-05-22

**Authors:** Haily Chen, Mercedes Montero-Vale, Kwabena Owusu-kyei, Alexandra Lara-Muñoz, Elisa Rubio-Garcia, Roger de Pedro-Jové, Elisabet Guiral, Maureen N. Chileshe, Julian Williams, Andreu Bofill, Mohamed Samai, Climent Casals-Pascual, Jordi Vila, Clara Menéndez

**Affiliations:** 1 Barcelona Institute for Global Health, Hospital Clinic-University of Barcelona, Barcelona, Spain; 2 Facultat de Medicina i Ciències de la Salut, Universitat de Barcelona (UB), Barcelona, Spain; 3 Investigación Biomédica en Red de Epidemiología y Salud Pública (CIBERESP), Madrid, Spain; 4 Department of Clinical Microbiology, Hospital Clínic, Barcelona, Spain; 5 Molecular CORE Facility, Hospital Clínic, Barcelona, Spain; 6 Centro de Investigación Biomédica en Red de Enfermedades Infecciosas (CIBERINFEC), Madrid, Spain; 7 College of Medicine and Allied Health Sciences, University of Sierra Leone, Freetown, Sierra Leone; 8 Manhiça Health Research Center (CISM), Manhiça, Mozambique; 9 Servicio de Salud Internacional, Hospital Clínic de Barcelona, Barcelona, Spain

**Keywords:** azithromycin, infants, macrolide resistance genes, pneumococcal carriage, Sierra Leone, *Streptococcus pneumoniae*

## Abstract

*Streptococcus pneumoniae* remains a major cause of child morbidity and mortality in sub-Saharan Africa, with increasing macrolide resistance mediated by *erm(B)* and *mef(A/E/I)*. Integrating phenotypic and sequencing-based approaches may improve antimicrobial resistance surveillance accuracy. This study assessed nasopharyngeal carriage and macrolide resistance of *S. pneumoniae* among young infants in Sierra Leone (November 2022–February 2023), nested within the ICARIA trial (NCT04235816) which evaluated azithromycin for child mortality reduction. Infants aged 6–10 weeks presenting for Penta-1 immunization before trial recruitment were enrolled. Two nasopharyngeal swabs were collected per infant. *S. pneumoniae* was detected by *lytA* PCR, azithromycin minimal inhibitory concentrations (MICs) were determined by E-test, and *erm(B)* and *mef(A/E)* were identified by PCR in isolates. A subset of paired samples underwent targeted amplicon sequencing for microbiome and resistome profiling. Carriage prevalence was 17.3% (162/936; 95% CI: 14.9–19.9%), with 45.7% (74/162) of isolates resistant to azithromycin (MIC ≥ 2 μg/mL). High-level resistance (MIC ≥ 64 μg/mL) was mainly mediated by *erm(B)* alone (53.8%) or in combination with *mef(A/E)* (38.5%), whereas all 22 moderately resistant isolates (MIC 2–48 μg/mL) carried *mef(A/E)* only. Among susceptible isolates, 3.8% (2/52) harbored *mef(A/E)* despite low MICs*.* Microbiome sequencing showed 96% concordance with *lytA* PCR for *S. pneumoniae* detection. Normalized resistome read counts for *erm(B)* and *mef(A/E)* were significantly higher in PCR-positive samples (*p* = 1.98 × 10^−9^ and *p* = 8.14 × 10^−7^). These findings provide the first estimates of nasopharyngeal *S. pneumoniae* carriage and macrolide resistance among infants in Sierra Leone, revealing a high prevalence of resistance. The results underscore the need to strengthen antibiotic stewardship, particularly in child survival programs with azithromycin. Large and longitudinal studies are also needed.

**Clinical Trial Registration**: ClinicalTrials.gov, NCT04235816.

## Introduction

*Streptococcus pneumoniae* commonly colonizes the upper respiratory tract from early life and, although often asymptomatic, can cause invasive infections including meningitis and pneumonia (Narciso et al., 2025). Despite widespread introduction of pneumococcal conjugate vaccines (PCVs) in the early 2000s, *S. pneumoniae* remains a leading cause of morbidity and mortality in sub-Saharan Africa (SSA), particularly among young children and individuals living with HIV. Although under-five (U5) mortality has declined by more than half over the past three decades, it remains high, with an estimated 4.9 million deaths globally in 2024, largely driven by lower respiratory infections (WHO, 2026). In a postmortem study of U5 children in low- and middle-income countries, *S. pneumoniae* was detected in 20% (14/69) of infection-attributable deaths (Baillie et al., 2025).

Young infants are particularly vulnerable to *S. pneumoniae* due to their immature immune systems, which limit their ability to effectively recognize and clear pathogens (Agnovia, 2025). Although U5 children have the highest reported carriage rates overall (Kwesi-Maliepaard et al., 2025), data on carriage in early infancy remain limited, particularly in Sierra Leone.

In Sierra Leone, the 13-valent pneumococcal conjugate vaccine (PCV13; serotypes 1, 3, 4, 5, 6A, 6B, 7F, 9V, 14, 18C, 19A, 19F, and 23F) was introduced in 2011 through the Expanded Program on Immunization (EPI), in line with World Health Organization (WHO) recommendations (Ministry of Health and Sanitation SL, 2014). The vaccine is administered in a three-dose schedule at approximately 6, 10, and 14 weeks of age. However, persistent health system challenges, further exacerbated by the Ebola outbreak and the COVID-19 pandemic, and limited laboratory diagnostic capacity have constrained surveillance of *S. pneumoniae*. Consequently, data on pneumococcal carriage in Sierra Leone remain scarce, limiting the evidence base needed to inform public health policy. As the first dose of PCV is administered at approximately 6 weeks of age, this timepoint provides a valuable opportunity to assess pneumococcal carriage prior to direct vaccine effects.

Antimicrobial resistance (AMR) in *S. pneumoniae* is an increasing global concern. Following the emergence of penicillin resistance, macrolides have been widely used as alternative treatments; however, increasing reports of clinical failure with azithromycin are of growing concern. In SSA, reliance on empirical treatment, treatment non-compliance, and the circulation of counterfeit and substandard drugs has contributed to the development of antibiotic resistance. Macrolide resistance is primarily mediated by ribosomal target modification via *erm(B)* and active drug efflux via *mef(A/E)* (Lohsen and Stephens, 2023). Geographically, *mef*(*A*) predominates in Europe, whereas *mef(E)* is more common in Africa, Asia, and the United States (Cornick and Bentley, 2012). The dual resistance genotype *erm(B)* + *mef(A/E)* confers higher levels of resistance and has been increasingly reported worldwide, including South Africa (Cornick and Bentley, 2012).

Next-generation sequencing (NGS) enables comprehensive characterization of the resistome and provides critical insights into the genetic basis of resistance and its transformation pathways (Hackman et al., 2024; Yang et al., 2022; Manyahi et al., 2023). However, discrepancies between genotypic detection and phenotypic resistance may arise due to regulatory mechanisms, serotype distribution, environmental conditions and epigenetic modifications (Cornick and Bentley, 2012). Integrating phenotypic and genotypic data is therefore essential for accurate resistance surveillance.

The main objectives of this study were to estimate the prevalence of nasopharyngeal *S. pneumoniae* carriage and macrolide resistance among young infants attending the 6-week EPI visit in Sierra Leone, allowing assessment prior to vaccination. In addition, we assessed the concordance between phenotypic and genotypic detection of macrolide resistance determinants.

## Methods

### Study design

This cross-sectional study was nested within the ICARIA trial (ClinicalTrials.gov identifier: NCT04235816) conducted in Sierra Leone, which evaluated the effect of azithromycin co-administered with routine EPI vaccines on all-cause mortality among 20,560 children. Azithromycin or placebo was administered at approximately 6 weeks, 9 months and 15 months during routine immunization visits (see Table 1 for the vaccination schedule). Detailed trial methods have been described elsewhere (Owusu-Kyei et al., 2024).

**Table 1:** Vaccination schedule of the expanded program on immunization in Sierra Leone in 2021 (Wassenaar et al., 2024).

Recommended timing of EPI contact	Intervention name	Detail
At birth	BCG	Bacillus Calmette–Guérin vaccine
	OPV 0	Oral polio vaccine
At 6 weeks	Penta 1	Diphtheria-Tetanus-Pertussis, Hepatitis B, *Haemophilus influenzae* type B vaccine 1st dose
	Pneumococcal 1	Pneumococcal conjugate vaccine (PCV13) 1st dose
	Rotavirus 1	Rotavirus vaccine 1st dose
At 10 weeks	OPV 2	Oral polio vaccine 2nd dose
	Penta 2	Diphtheria-Tetanus-Pertussis, Hepatitis B, *Haemophilus influenzae* type B vaccine 2nd dose
	Pneumococcal 2	Pneumococcal conjugate vaccine (PCV13) 2nd dose
	Rotavirus 2 vaccine	Rotavirus vaccine 2nd dose
	PMC 1	Perennial malaria chemoprevention tablet 1st dose
At 14 weeks	OPV 3	Oral polio vaccine 3rd dose
	Penta 3	Diphtheria-Tetanus-Pertussis, Hepatitis B, *Haemophilus influenzae* type B vaccine 3rd dose
	Pneumococcal 3	Pneumococcal conjugate vaccine (PCV13) 3rd dose
	IPV	Inactivated polio vaccine
	PMC 2	Perennial malaria chemoprevention tablet 2nd dose
At 6 months	Vitamin A	Vitamin A supplement
At 9 months	Yellow fever	Yellow fever vaccine
	MCV 1	Measles-containing-vaccine 1st dose
	PMC 3	Perennial malaria chemoprevention tablet 3rd dose
At 12 months	De-worming	Mebendazole (500 mg) or albendazole (400 mg)
	Vitamin A	Vitamin A supplement
At 15 months	MCV 2	Measles-containing-vaccine 2nd dose

Between November 2022 and February 2023, infants aged 6–10 weeks attending selected U5 clinics for their first PCV13 vaccine in Bombali and Tonkolili districts were enrolled. Health facilities were selected based on vaccination coverage and proximity to the central laboratory to ensure sample transport and storage feasibility.

Infants were eligible if they were between 6 and 10 weeks of age, eligible to receive PCV13 according to EPI guideline but have not received PCV13 vaccine, residing within the health facility catchment areas, and weighed ≥2.5 kg. Infants were excluded if they had resided outside the catchment area in the preceding 12 months or presented with signs of acute illness that precluded vaccination at the visit.

After written parental consent was given, two nasopharyngeal swabs (NPS) were collected per infant, and demographic data were collected using a questionnaire implemented on REDCap.

### Nasopharyngeal sample collection

Two NPS were collected per participant using sterile calcium alginate swabs (Deltalab, Barcelona, Spain). One was placed in skim milk-tryptone-glucose-glycerin (STGG) (O’Brien et al., 2001) medium for isolate-based phenotypic and genotypic analysis, and the other in DNA/RNA Shield medium (Zymo Research Corporation, Irvine, CA, United States) for whole-sample sequencing. Samples were transported to the local laboratory within 4 h in temperature-controlled containers. STGG samples were stored at −80 °C and DNA/RNA Shield samples at −20 °C. STGG samples were shipped at −20 °C, while DNA/RNA Shield samples at ambient temperature to the Hospital Clínic Microbiology Laboratory in Barcelona for downstream analysis.

### Laboratory methods

#### Isolate identification and antimicrobial susceptibility testing of *Streptococcus pneumoniae*

Nasopharyngeal samples preserved in STGG were enriched in Todd-Hewitt broth and plated on Columbia nalidixic acid agar (Becton Dickinson GmbH, Germany) to isolate *S. pneumoniae*. Presumptive pneumococcal colonies were identified based on colony morphology, MALDI-TOF mass spectrometry and optochin susceptibility test (Becton Dickinson GmbH).

Molecular confirmation was performed by *lytA* PCR (Conda Lab, Spain), with primers listed in Table 2. Optochin-susceptible but *lytA*-negative isolates underwent species differentiation within the *Streptococcus mitis* group (SMG) using species-specific PCR assays to distinguish *S. pneumoniae* from *S. pseudopneumoniae*, with housekeeping gene as controls (Department of Clinical Microbiology, Bellvitge Hospital, Barcelona, Spain) (Basheer et al., 2024).

**Table 2:** Sequences of primers genes used in PCR.

Gene(s)	Primer sequences (5′ to 3′)	Amplicon size (bp)	References
*lytA*	GGA GTA GAA TAT GGA AAT TAA TGT GCT GCA TAG GTC TCA GCA TTC CAA	263	Gillespie et al. (1994)
*SPN0001*	AATATCTGAAGATGCTCATTCTACAATT ATAAGGTTTACCGTCAATAATACGCAG	154	Croxen et al. (2018)
*SPPN_RS10375*	CTAATTGCTACTGCTATTTCCGGTG CTGATACCTGCAACAAAAATCGAAG	402	Garriss et al. (2019)
*map*	GCWGACTCWTGTTGGGCWTATGC TTARTAAGTTCYTTCTTCDCCTTG	459	Bishop et al. (2009)
*guaA*	ATYCARTTYCACCCMGAAGT CWGGNCCWGGRAATGGTTG	679	Bishop et al. (2009)
*erm*(B)	TGGTATTCCAAATGCGTAATG CTGTGGTATGGCGGGTAAGT	745	Malhotra-Kumar et al. (2005)
*mef*(A/E)	CAATATGGGCAGGGCAAG AAGCTGTTCCAATGCTACGG	317	Bishop et al. (2009)

Azithromycin susceptibility was determined by E-test (bioMérieux, Spain), and minimum inhibitory concentrations (MICs) were interpreted according to Clinical and Laboratory Standards Institute guidelines (CLSI M100, 36th edition, 2026), using > 2 μg/mL defined as resistant (Clinical and Laboratory Standards Institute, 2012).

#### Isolate-based genotyping of macrolide resistance determinants

PCR screening for *erm(B)* and *mef(A/E)* was performed on all azithromycin-resistant isolates and a subset of susceptible isolates (Malhotra-Kumar et al., 2005). Primers are provided in Table 2.

Resistant isolates lacking detectable macrolide resistance genes by PCR underwent whole-genome sequencing using Oxford Nanopore Technology (ONT). Genomic DNA was extracted using the cetyltrimethylammonium bromide (CTAB) methodology described by Pinzauti et al. (2022), and the libraires were prepared using the Rapid Barcoding Kit (SQK-RBK114; Oxford Nanopore Technologies). Long-read data were assembled *de novo* and screened for antimicrobial resistance genes using a custom Nextflow pipeline (AMRmicrobiology, 2025).

#### Whole-sample microbiome and resistome sequencing

**Figure 1:**
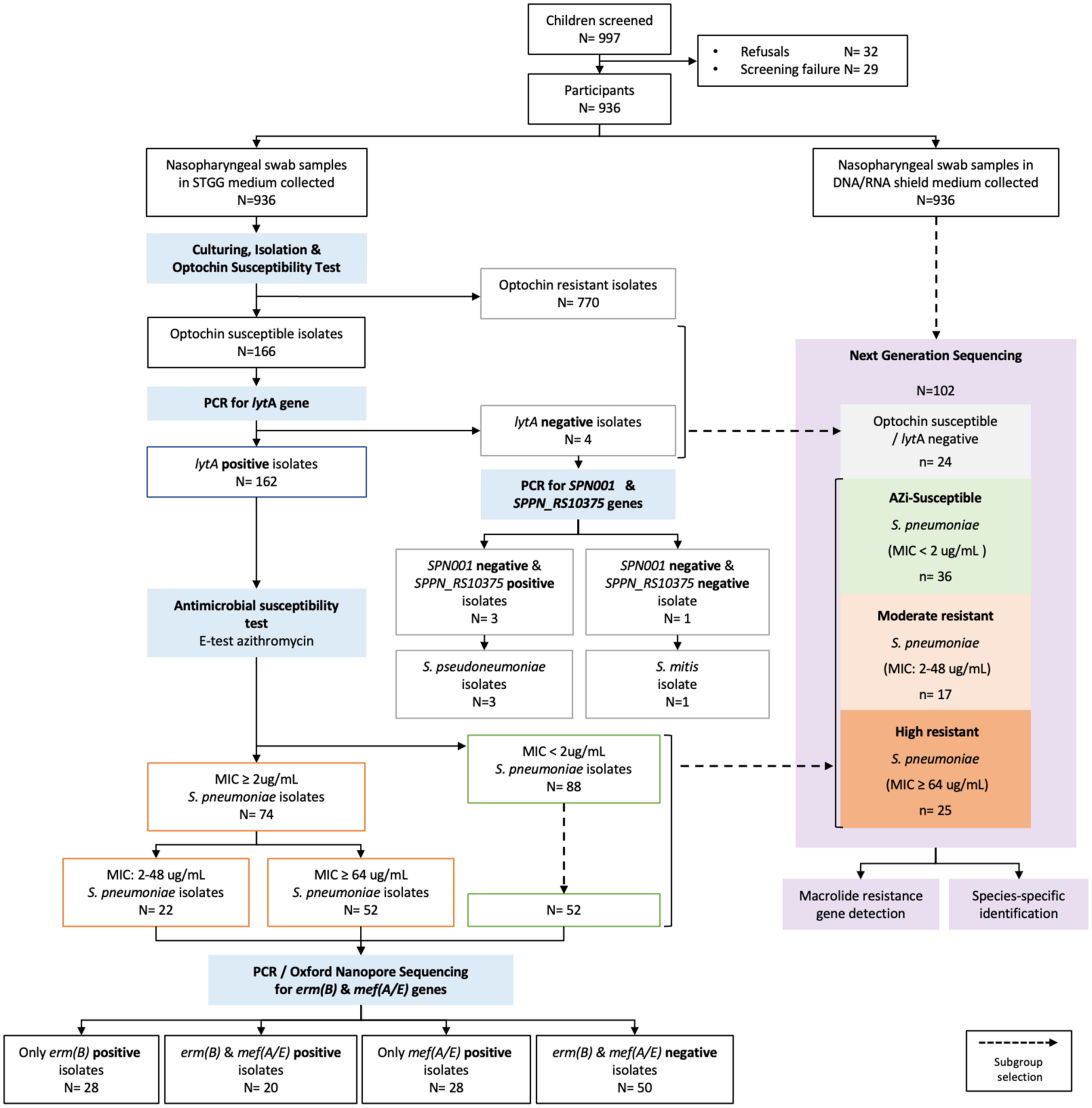
Workflow for the identification and characterization of *Streptococcus pneumoniae* isolates and macrolide resistance. The schematic summarizes participant enrollment, sample processing, and laboratory analysis workflow. Detailed procedures for sample collection, culture conditions, and molecular assays are provided in the Supplementary material. Left branch: Nasopharyngeal swabs (NPS) in STGG broth were cultured, and optochin-susceptible isolates were confirmed as *S. pneumoniae* by *lytA* PCR. Azithromycin-resistant isolates along with a subset of sensitive isolates were further characterized for the resistance determinants *erm* ***(B)*** and *mef(A/E)*. Right branch: A subset of matched NPS samples preserved in DNA/RNA Shield underwent next-generation sequencing (NGS) to enable comprehensive detection of macrolide resistance genes and species identification. MIC, Minimum Inhibitory Concentration; CLSI, Clinical and Laboratory Standards Institute.

A subset of in DNA/RNA Shield-preserved NPS samples was selected for sequencing based on azithromycin MIC values of the corresponding STGG samples. Samples were randomly selected across resistance categories (azithromycin-susceptible, moderately resistant, highly resistant, and negative, as shown in Figure 1) to ensure balanced representation.

DNA was extracted from swabs and transport medium using a KingFisher Flex automated extraction platform with Microbiome MagMAX Extraction Kit (Life Technologies, CA, United States). Bacterial community composition and resistome were characterized in 102 samples using a targeted a multiplex amplicon-based panel (Ion AmpliSeq™ Pan-Bacterial Research Panel, Thermo Fisher Scientific).

Bioinformatic analysis was performed using the PanBacterialAnalysis plugin in the Torrent Suite software for microbiome and resistome profiling. The pipeline includes automated quality control, read filtering and assignment to reference targets. Sequencing data were evaluated based on overall read depth and target assignment prior to downstream analyses.

For Pool 1, read counts mapping to antimicrobial resistance genes (ARGs) and bacterial species were annotated using the Comprehensive Antibiotic Resistance Database (CARD). Read counts for macrolide resistance genes were aggregated per sample. For Pool 2, reads were aligned to the Greengenes 16S rRNA reference database for microbiome profiling. All read counts were normalized to total reads per sample to account for differences in sequencing depth.

#### Comparison of whole-sample sequencing with isolate-based genotyping

Whole-sample microbiome and resistome sequencing results were compared with isolate-based *S. pneumoniae* detection and macrolide resistance genotyping. Detected resistance genes were evaluated against isolate-based findings, and read counts were normalized to sequencing depth prior to downstream analyses.

Detailed laboratory procedures, sequencing workflows and bioinformatic methods are provided in the Supplementary material.

### Statistical analysis

The sample size was calculated to detect a difference in azithromycin-resistant *S. pneumoniae* prevalence between 3.0% in the placebo group and 9.0% in the azithromycin group of the ICARIA trial. Assuming 75% nasopharyngeal carriage, a two-sided significance level of 0.05, and 80% power, 327 children per group were required (Adubra et al., 2023). To account for an estimated 30% samples unsuitable for laboratory analysis, the target sample size was increased to 467 per group.

Participant characteristics and prevalence were summarized using descriptive statistics. Continuous variables were reported as means with standard deviations (SD), and categorical variables as frequencies. Seasons were classified as rainy (May–October) or dry (November–April). Caretaker literacy was defined as the ability to read and write a full sentence. Weight-for-age Z-scores (WAZ) were calculated using WHO standards; underweight was defined as WAZ < −2 (WHO, 2025a,b).

Associations between carriage and participant characteristics were assessed using logistic regression. Multivariable models included district, season, caretaker marital status and literacy, child sex, age, and nutritional status. Analyses were conducted in Stata version 18.

Concordance between whole-sample sequencing and isolate-based detection was assessed by comparing normalized *S. pneumoniae* read counts with *lytA* PCR results. Sequencing positivity was defined using the *cutpoint* package in R to identify an empirically derived threshold maximizing classification accuracy. For macrolide resistance genes, normalized read counts for *erm(B)* and *mef(A/E)* were compared between PCR-positive and PCR-negative samples using the Mann–Whitney U test. Statistical significance was set at *p* < 0.05.

### Ethical considerations

The study protocol and informed consent were approved by the Sierra Leone Ethics and Scientific Review Committee and the Hospital Clinic Research Ethics Committee (Barcelona, Spain) (Registration No.: HCB/2020/0173). Written informed consent was obtained from caretakers prior to participation. The funder had no role in the design, data collection, analysis, interpretation, or manuscript preparation.

## Results

### Characteristics of study participants

Between November 2022 and February 2023, 997 infants were screened at four U5 clinics in the Bombali and Tonkolili districts of Northern Sierra Leone. Thirty-two caretakers declined to participate and 29 infants were ineligible, resulting in 936 enrolled infants, each providing two NPS were collected. Participant characteristics are summarized in Table 3.

**Table 3:** Characteristics of study participants and prevalence of *S. pneumoniae* carriage.

Participant characteristics		*S. pneumoniae* positive *n* (*n*/*N*%)	*N* (%)
District	Bombali	102 (16.0%)	636 (67.9%)
	Tonkolili	60 (20.0%)	300 (32.1%)
Seasonality	Rainy	4 (9.5%)	42 (4.5%)
	Dry	158 (17.7%)	894 (95.5%)
Caretaker’s marital status	Not married	36 (15.1%)	239 (25.5%)
	Married or in union	124 (18.0%)	688 (73.5%)
	NA	2 (22.2%)	9 (1.0%)
Caretaker’s education level	Never attended school	52 (22.2%)	234 (25.0%)
	Attended school	108 (15.6%)	693 (74.0%)
	NA	2 (22.2%)	9 (1.0%)
Caretaker’s literacy	Illiterate	121 (19.2%)	631 (67.4%)
	Literate	39 (13.2%)	296 (31.6%)
	NA	2 (22.2%)	9 (1.0%)
Child sex	Male	71 (15.1%)	471 (50.3%)
	Female	91 (19.6%)	465 (49.7%)
Child age (weeks, SD)*		6.7 ± 0.8	6.6 ± 0.7
Underweight children	No	149 (17.5%)	850 (90.8%)
	Yes	13 (15.2%)	86 (9.2%)

Summary of participant characteristics and the number and proportion colonized with *S. pneumoniae*, stratified by district, season, caretaker sociodemographic factors (marital status, education, and literacy), and child characteristics (sex, age, and underweight status). *Child age is presented as mean (SD).

Most infants resided in Bombali district (636; 68.0%) and were recruited during the dry season (894; 95.5%). The mean age at enrollment was 6.6 ± 0.7 weeks; 50.3% (471) were male and 9.2% (86) underweight. Most caretakers were married (73.5%, 688) and had attended school (74.0%, 693), while 31.6% (296) were classified as literate.

### Social determinants of *Streptococcus pneumoniae* carriage

Associations between participant characteristics and nasopharyngeal carriage are presented in Table 4. In univariate analyses, higher caretaker education (OR: 0.65; 95% CI: 0.45–0.94; *p* = 0.02) and literacy (OR: 0.64; 95% CI: 0.43–0.95; *p* = 0.03) were associated with lower odds of carriage, while increasing infant age was also associated with higher odds (OR: 1.53; 95% CI: 1.08–2.18; *p* = 0.02).

**Table 4:** Association between potential risk factors and pneumococcal colonization.

Characteristics		Crude odds ratio (95% CI)	*P*-value	Adjusted odds ratio (95% CI)	*P*-value
District (*n* = 936)	Bombali	1		1	
	Tonkolili	1.31 (0.92, 1.86)	0.14	1.35 (0.94, 1.94)	0.10
Season (*n* = 936)	Rainy	1		1	
	Dry	2.04 (0.72, 5.80)	0.18	1.95 (0.67, 5.66)	0.22
Caretakers’ education (*n* = 903)	Never attended school	1		–	–
	Attended school	**0.65 (0.45, 0.94)**	**0.02**	–	–
Caretaker’s literacy (*n* = 927)	Illiterate	1		1	
	Literate	**0.64 (0.43, 0.95)**	**0.03**	**0.60 (0.41, 0.89)**	**0.01**
Caretaker’s marital status (*n* = 927)	Not married	1		1	
	Married	1.24 (0.83, 1.86)	0.30	1.15 (0.76, 1.74)	0.50
Child sex (*n* = 936)	Male	1		1	
	Female	1.37 (0.97, 1.93)	0.07	1.30 (0.92, 1.85)	0.14
Age in week (*n* = 936)	<6.5 week	1		1	
	>6.5 week	**1.53 (1.08, 2.18)**	**0.02**	1.40 (0.98, 2.00)	0.07
Underweight (WHO) (*n* = 936)		0.84 (0.45, 1.55)	0.57	0.95 (0.50, 1.79)	0.87

Crude and adjusted odds ratios from univariate and multivariable logistic regression analyses assessing potential risk factors for *Streptococcus pneumoniae* colonization. CI, confidence interval. Bolded values are of significant difference.

In multivariable analysis, caretaker literacy remained independently associated with reduced odds of *S. pneumoniae* carriage (adjusted OR: 0.60; 95% CI: 0.41–0.89; *p* = 0.01).

### Isolate-based genotyping detection of *Streptococcus pneumoniae* and macrolide resistance

Participants enrollment and laboratory workflow are shown in Figure 1. Of the 936 NPS analyzed, 162 (17.3%; 95% CI: 14.9–19.9%) were positive for *S. pneumoniae,* defined by optochin susceptibility, PCR positivity for *lytA* and *SPN001*, and PCR negativity for *SPPN_RS10375*. Of four optochin-susceptible but *lyt*A-negative isolates, one was identified as *Streptococcus mitis* and three as *Streptococcus pseudopneumoniae.*

**Figure 2:**
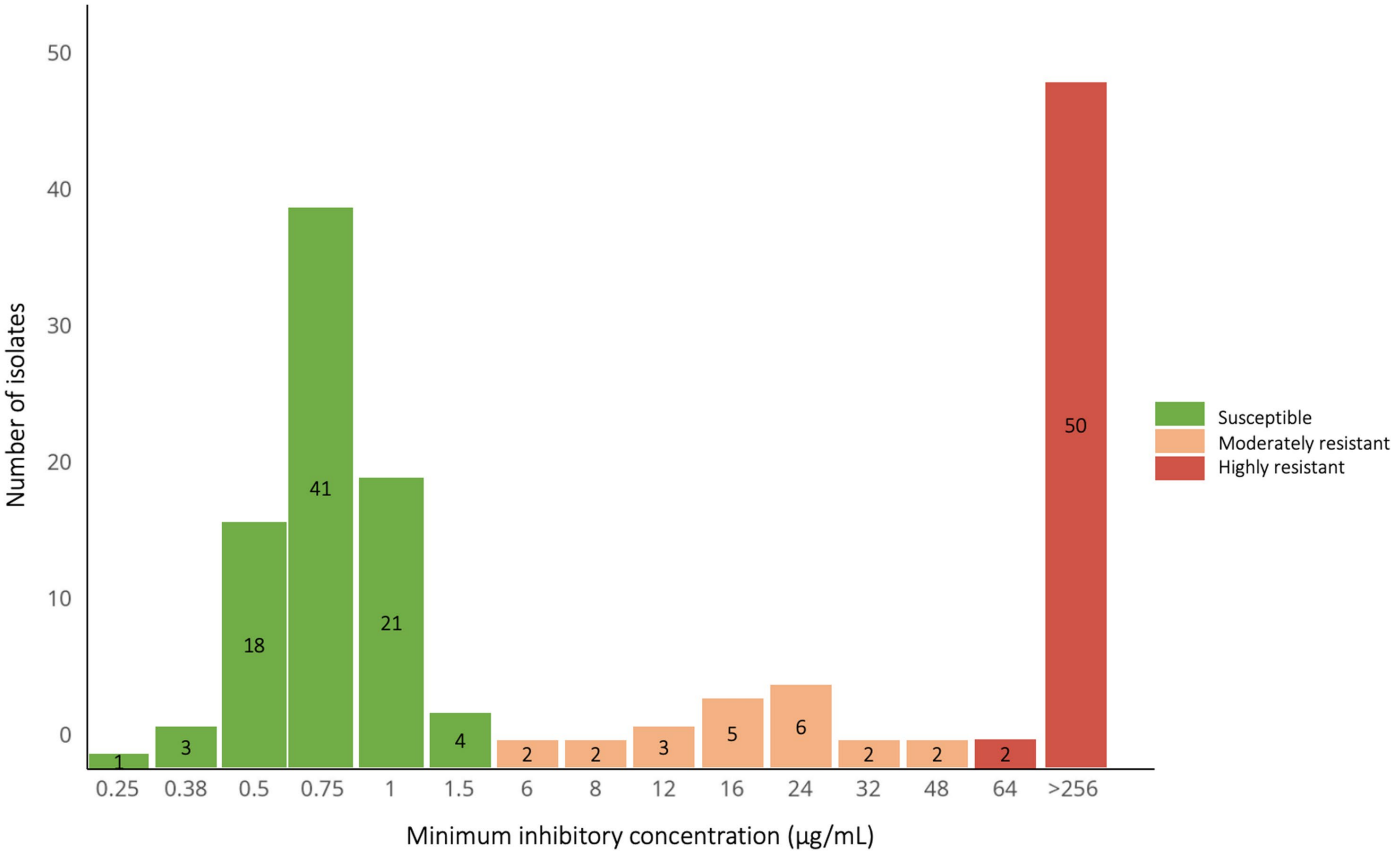
Distribution of azithromycin minimum inhibitory concentration (MIC) values for *Streptococcus pneumoniae* isolates (*N* = 162) across author-defined resistance categories. MIC values were determined by E-test. Isolates were classified as susceptible (MIC < 2 μg/mL) per Clinical and Laboratory Standards Institute (CLSI) guidelines. Resistant isolates (MIC ≥ 2 μg/mL) were further stratified by the authors into moderately resistant (MIC: 2–48 μg/mL) and highly resistant (MIC ≥ 64 μg/mL) categories for analysis.

Among confirmed *S. pneumoniae* isolates, 88 (54.3%) were azithromycin-susceptible (MIC < 2 μg/mL), while 74 (45.7%) resistant (MIC ≥ 2 μg/mL) (Figure 2). Of the resistant isolates, 52 (70.3%) exhibited high-level resistance (MIC ≥ 64 μg/mL) and 22 (29.7%) moderate resistance (MIC 2–48 μg/mL). If isolates with MICs of 0.75–1.5 g/mL (*n* = 66) were classified as resistant, the overall resistance rate would increase to 86%.

**Figure 3:**
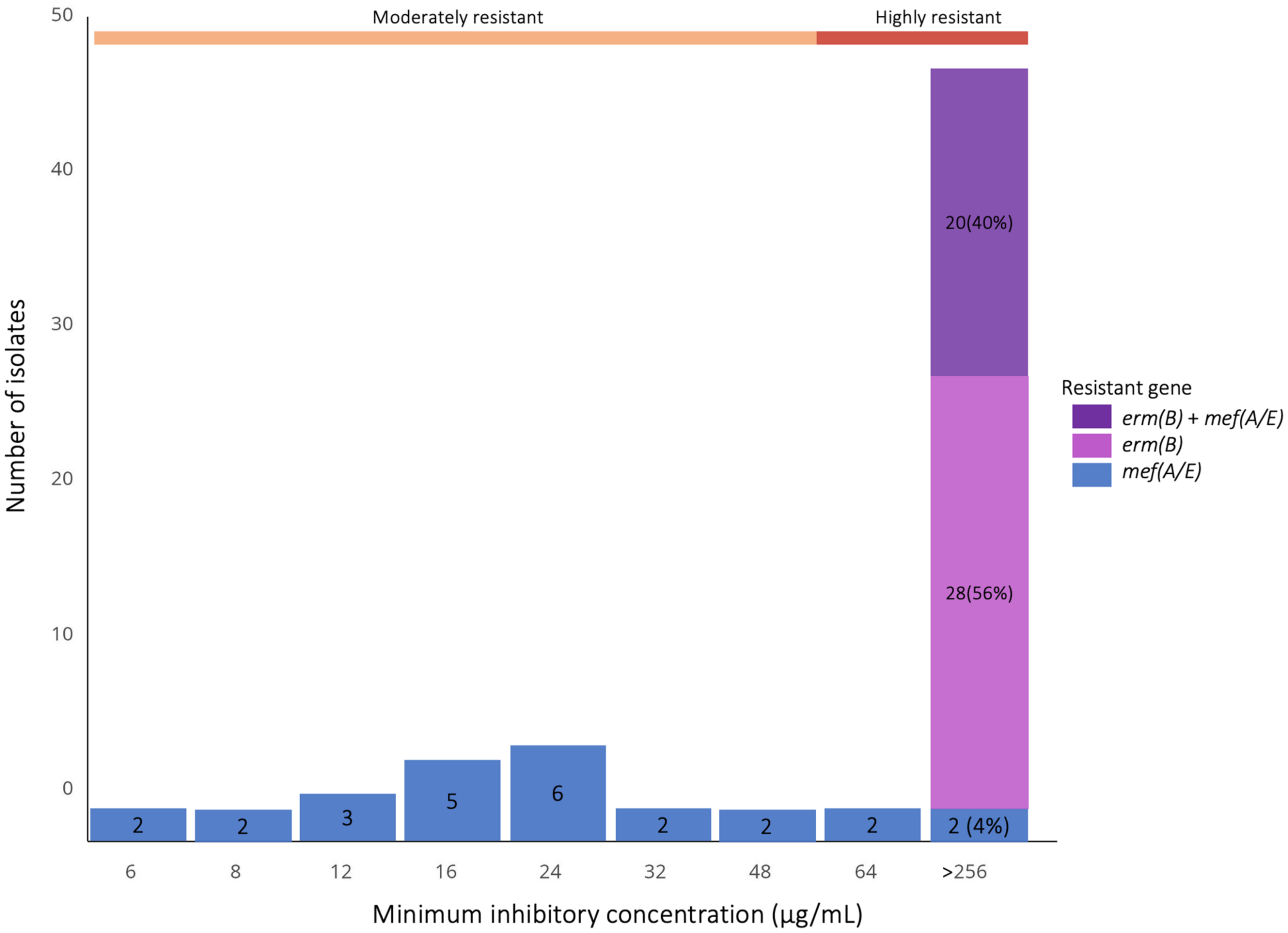
Prevalence of *erm*(B) and *mef*(A/E) resistance genes among azithromycin-resistant *S. pneumoniae* isolates (*N* = 74), stratified by MIC value. The bar chart shows the frequencies of resistance gene profiles—(*erm*(B) only, *mef*(A/E) only, or both)—across defined MIC values for azithromycin-resistant isolates.

Isolate-based genotyping showed that among highly resistant isolates, 28 (53.8%) carried *erm(B)* alone, four (7.7%) *mef(A/E)* alone, and 20 (38.5%) both genes. All 22 moderately resistant isolates harbored *mef(A/E)* exclusively. Among 52 azithromycin-susceptible isolates tested, two carried *mef(A/E)* (Figure 3); their MICs were 0.75 and 1.5 g/mL, respectively.

### Whole-sample detection of *Streptococcus pneumoniae* and macrolide resistance

**Figure 4:**
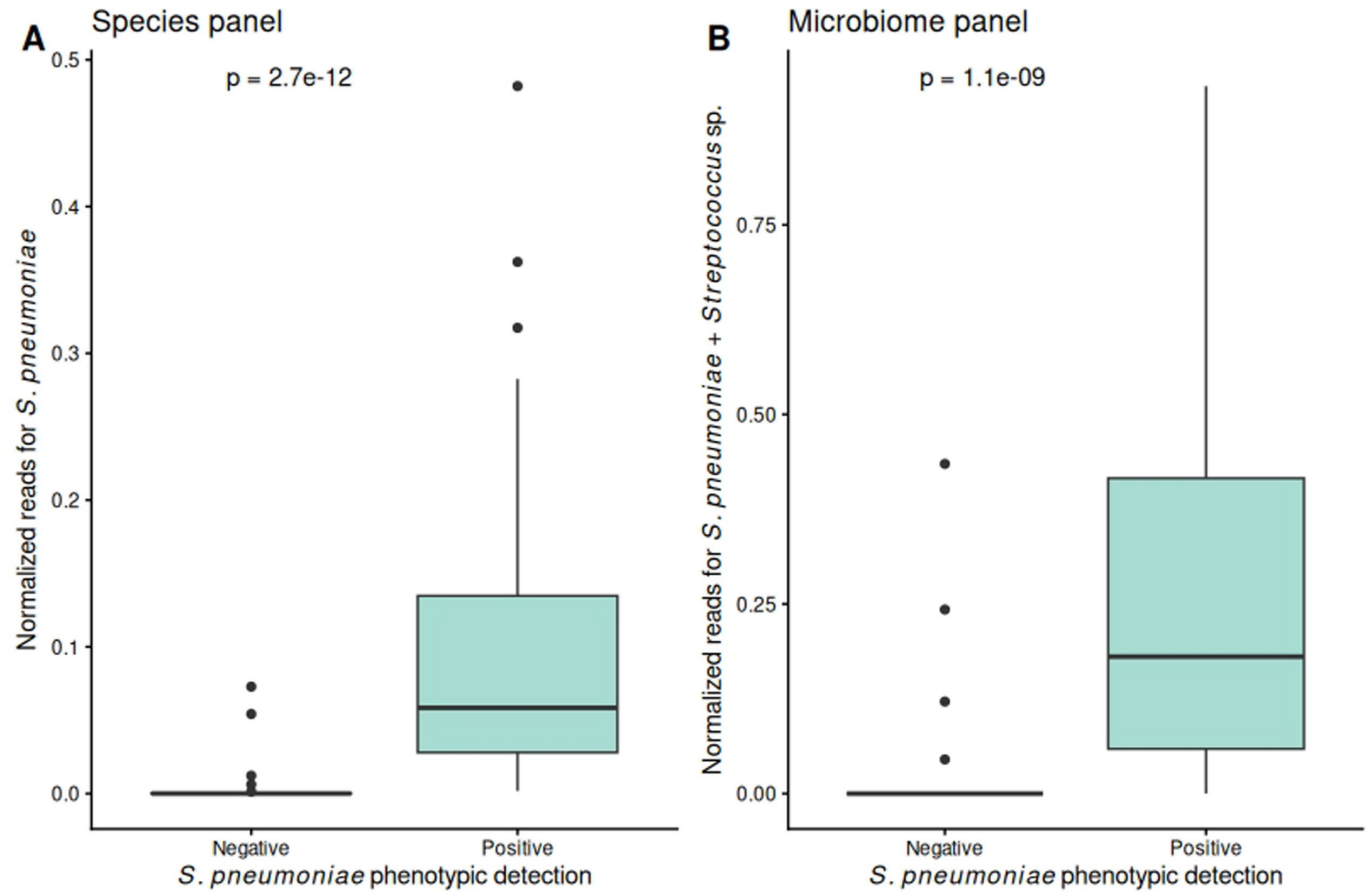
Correlation between isolate-based and whole-sample detection of nasopharyngeal *S. pneumoniae*. **(A)** The species panel includes amplicons targeting bacterial as well as antimicrobial resistance genes (ARGs) for resistome characterization. **(B)** Microbiome panel comprises amplicons targeting variable regions of the 16S rRNA to profile the broader microbial community.

Amplicon sequencing differentiated *S. pneumoniae-*positive and -negative samples based on normalized read counts for *S. pneumoniae* (Species panel, *p* = 2.7 × 10^−12^) and the *Streptococcus* genus (Microbiome panel, *p* = 1.1 × 10^−9^) (Figure 4). A data-driven threshold of 0.0018 normalized reads in the Species panel achieved 96% concordance with phenotypic and PCR-based detection in paired STGG samples.

**Figure 5:**
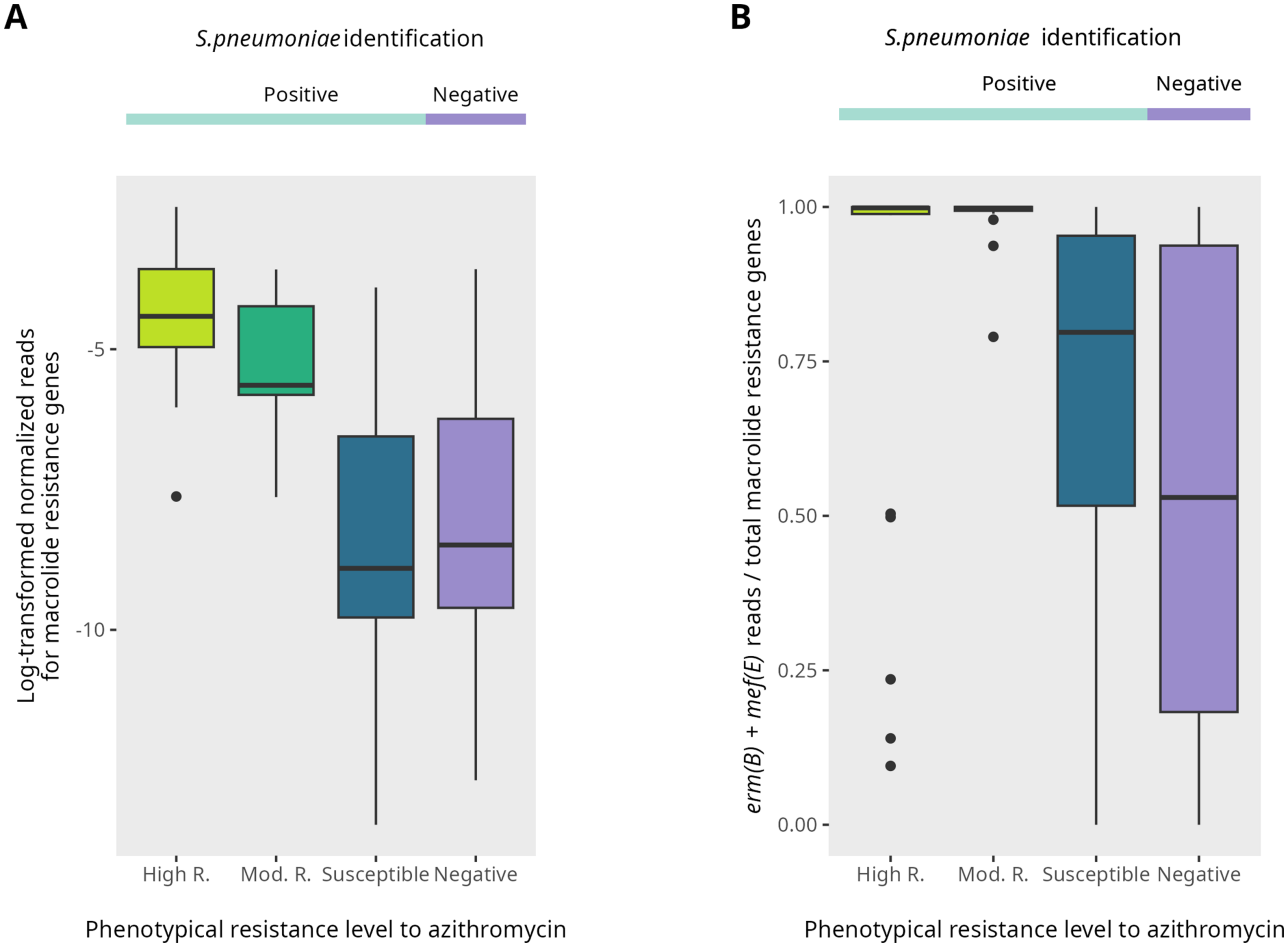
Distribution of resistome macrolide resistance across isolate-based genotyped resistance categories. **(A)** Total macrolide resistance burden, represented by log-transformed normalized read counts of all macrolide resistance genes detected by the Ion AmpliSeq™ Pan-Bacterial Research Panel in DNA from nasopharyngeal swabs (NPS). Genes were annotated using the Comprehensive Antibiotic Resistance Database (CARD). Log-transformed median (IQR) values by resistance category were: High resistant −4.41 (−4.96 to −3.57); Moderate resistant, −5.64 (−5.81 to −4.23); Sensitive, −8.90 (−9.78 to −6.55); and Negative, −8.49 (−9.61 to −6.24). **(B)** *Streptococcus pneumoniae*-specific macrolide resistance, shown as the relative abundance of *erm*(B) and *mef*(E) genes. Values are expressed as the proportion of combined *erm*(B) and *mef*(E) read counts relative to the total macrolide resistance gene reads. Median (IQR) values by resistance category were: High resistant, 0.998 (0.989–1.000); Moderate resistant, 0.998 (0.994–1.000); Sensitive, 0.797 (0.516–0.953); and Negative, 0.530 (0.182–0.937).

Resistome sequencing showed alignment between normalized read counts for macrolide resistance genes and phenotypic resistance levels, with significant differences across resistance groups (Kruskal–Wallis *p* = 2.046 × 10^−9^) (Figures 1, 5A). In samples carrying highly resistant isolates, the proportion of *erm(B)* and *mef(A/E)* reads relative to total macrolide resistance genes approached 1 and declined progressively across lower resistance categories (Kruskal–Wallis *p* = 2.09 × 10^−6^) (Figure 5B).

### Concordance between whole-sample sequencing and isolate-based genotyping

Agreement between whole-sample resistome sequencing and isolate-based PCR detection of *erm(B)* and *mef(A/E)* was evaluated in 75 paired DNA/RNA Shield–preserved samples and STGG-preserved samples (including phenotypically resistant and susceptible isolates). Normalized sequencing read counts were significantly higher in PCR-positive than PCR-negative samples for both *erm(B)* (mean and IQR 0.0051 [0.0037–0.0093] vs. 0.0000 [0.0000–0.0001]; Mann–Whitney *p* = 1.98 × 10^−9^) and *mef(A/E)* (mean and IQR 0.0041 [0.0026–0.0097] vs. 0.0001 [0.0000–0.0010]; *p* = 8.14 × 10^−7^).

Sequencing positivity was defined as ≥ 20 raw reads mapping to the target genes, a conservative threshold set above background signal. Using this criterion, discordance was observed in 29.3% of the samples for *erm(B)* and 45.3% for *mef(A/E).* All *erm(B)-*discordant samples were sequencing-positive but PCR-negative. For *mef(A/E),* 32 samples were sequencing-positive but PCR-negative, and two were sequencing-negative but PCR-positive.

Whole genome sequencing data available for two discordance isolates from prior ONT-based resistance analysis. In both cases, PCR-negative isolates were confirmed sequencing-positive, one for *erm(B)* and one for *mef(A/E),* consistent with whole-sample resistome findings.

## Discussion

This study provides the first estimates of nasopharyngeal *S. pneumoniae* carriage and associated macrolide resistance among young infants in Sierra Leone, more than a decade after PCV13 introduction and within the context of a large-scale clinical trial evaluating azithromycin for child mortality reduction. We also present the first detailed characterization of macrolide resistance mechanisms in the country, integrating phenotypic isolate-based methods with whole-sample resistome sequencing.

At 6 weeks of age, prior to receiving both the first dose of PCV13 and the ICARIA intervention (azithromycin or placebo), pneumococcal carriage prevalence was 17.3% (95% CI: 14.9–19.9%) and nearly half of them (45.7%) were resistant to macrolide. These findings reflect substantial community-level antibiotic pressure in the absence of direct azithromycin exposure through the trial.

Nasopharyngeal *S. pneumoniae* colonization is a prerequisite for invasive pneumococcal disease, which contributes substantially to morbidity and mortality among U5 children. Acquisition typically occurs in early infancy through household contact (Usuf et al., 2018). Previous studies have reported a median age of first acquisition of 4.7 weeks in The Gambia (Hill et al., 2008), 5.5 weeks in Kenya (Tigoi et al., 2012), and 6.5 weeks in the Thailand-Myanmar border region (Turner et al., 2012), underscoring that early infancy represents a critical window for pneumococcal transmission and prevention.

The carriage prevalence of 17.3% at 6 weeks lies at the lower end of estimates reported from other low- and middle-income settings, which range from 20% among 6-week-olds in South Africa (2012–2014) to 65.6% among 1-month-olds in Papua New Guinea (2011–2016) (Dube et al., 2018; Temple et al., 2023; Orami et al., 2023; Kawade et al., 2023; Prayitno et al., 2021; Usuf et al., 2019). This relatively low prevalence likely reflects indirect (herd) effects of PCV13, given sustained high vaccine coverage in Sierra Leone since its introduction in 2011 (average three-dose coverage: 89.8%) (Wassenaar et al., 2024). Although maternal immunity may contribute to partial protection in early infancy (Agnovia, 2025), herd immunity is likely the primary driver. Young infants may therefore serve as a potential sentinel population for monitoring indirect vaccine effects, as their carriage reflects community transmission prior to direct immunization.

In addition to vaccination, pneumococcal carriage is influenced by household and environmental factors, including crowding, number of siblings, smoking exposure, and indoor air pollution (Dherani et al., 2022; Beissegulova et al., 2024). In our study, higher caregiver literacy and education were associated with lower odds of carriage, consistent with evidence that socioeconomic factors influence early life colonization risk through differences in hygiene practices, household crowding, healthcare-seeking behaviors, and antibiotic use (Bhusal and Khanal, 2022).

Despite the relatively low carriage prevalence, 45.7% of the isolates were resistant to azithromycin at baseline. Although infants had not been directly exposed to azithromycin through the trial, macrolides are widely used in primary care settings, and resistance likely reflects cumulative antibiotic pressure in the community, including over-the-counter access (Kanu et al., 2021). High-level resistance was predominantly mediated by *erm(B)* alone or in combination with *mef(A/E),* consistent with ribosomal methylation as the primary mechanism of high-level resistance, whereas moderate resistance was exclusively associated with *mef(A/E)*-mediated efflux. The detection of macrolide resistance genes in a small number of phenotypically susceptible isolates highlights the limitations of phenotypic testing alone (Cornick and Bentley, 2012). Although EUCAST defined an epidemiological cut-off of 0.25 g/mL for azithromycin in *S. pneumoniae* to define the upper end of the wild-type MIC distribution without phenotypically detectable acquired resistance mechanisms, isolates with MICs above this threshold are considered non–wild-type and are likely to harbor acquired resistance mechanisms. In our study, only two of the 88 isolates with MICs between 0.25 and 1.5 μg/mL carried a detectable resistance mechanism. The high prevalence of macrolide resistance determinants (*erm(B)* and *mef(A/E)*) underscores the need to strengthen antibiotic stewardship in Sierra Leone (Kanu et al., 2021).

Microbiome sequencing showed 96% agreement with phenotypic and PCR-based detection, and normalized read counts closely corresponded with PCR results for macrolide resistance genes. These findings support the use of targeted amplicon sequencing to complement antimicrobial resistance surveillance, particularly in settings where culture-based isolation or preservation of viable isolates is challenging. Discordance between sequencing and isolate-based PCR for *erm(B)* and *mef(A/E)* likely reflects differences between whole-sample and single-isolate detection. Sequencing-positive but isolate PCR-negative results may indicate resistance genes present in in other members of the nasopharyngeal microbiota (Varaldo et al., 2009). Conversely, PCR-positive but sequencing-negative findings may occur when resistance genes are present at low abundance due to microbial competition or amplification bias during amplicon-based resistome characterization.

Accurate species-level identification within the SMG remains challenging. Even advanced diagnostic methods such as MALDI-TOF mass spectrometry and 16S rRNA gene sequencing show limited discriminatory capacity (Werno et al., 2012; Ikryannikova et al., 2013), and traditional phenotypic methods showed inconsistent performance (Croxen et al., 2018; Rolo et al., 2013; Arbique et al., 2004; Garriss et al., 2019). Combining phenotypic and molecular methods likely improved the specificity of pneumococcal identification and confidence in carriage estimates.

Several limitations of the study should be considered. First, only a limited number of colonies were selected during culture, and serotyping was not performed, which limits interpretation of vaccine-type versus non-vaccine-type carriage. Second, sequencing was performed on a subset of samples due to cost constraints, although these were selected across resistance profiles. Third, study sites were selected based on proximity to the central laboratory within two districts in Sierra Leone to preserve sample integrity, which may limit generalizability to more remote areas and other parts of the country. Fourth, the study was conducted during the dry season (November 2022–February 2023) and may not reflect seasonal variation in pneumococcal transmission. Finally, the cross-sectional design provides a snapshot of carriage and resistance but does not allow assessment of acquisition, persistence, or temporal trends.

In conclusion, although *S. pneumoniae* carriage was relatively low in infants as young as 6 weeks of age, likely reflecting indirect effects of PCV13 prior to direct vaccination. The high prevalence of macrolide resistance among carried strains raises important concerns for empirical antibiotic use. These findings underscore the need to strengthen antibiotic stewardship and support the integration of genomic surveillance in sentinel sites to guide targeted interventions and optimize treatment strategies in high-burden populations in low- and middle-income countries. This is particularly important in the context of mass azithromycin administration programs being implemented in several countries in SSA to improve child survival (REACH, 2025). Larger and longitudinal studies are needed to provide insight into the development of carriage.

## Data Availability

The datasets presented in this article can be found here: https://www.ncbi.nlm.nih.gov, accession number, PRJNA1453221.
