# Supplementary material for: Early-life carriage and antibiotic resistance of Streptococcus pneumoniae in infants from Sierra Leone
Source: Front Microbiol. 2026 May 22;17:1822296. doi: 10.3389/fmicb.2026.1822296 (PMC13238436; doi:10.3389/fmicb.2026.1822296)
Supplement: Supplementary file 1 [file Data_Sheet_1.DOCX]

**Supplemental material**

**S1. Phenotypic characterization of *Streptococcus* isolates**

STGG-preserved nasopharyngeal swab (NPS) samples were thawed on ice and vortexed for at least 20 s before enrichment in Todd Hewitt Broth at a 1:10 dilution. Enrichment cultures were incubated for 4 h at 37 °C in 5% CO₂ and subsequently inoculated onto Columbia nalidixic acid (CNA) agar plates (Becton Dickinson GmbH, Heidelberg, Germany). Plates were incubated at 37 °C in 5% CO₂ for 24 h and examined for small, grey, α-hemolytic, glistening colonies consistent with *Streptococcus* morphology.

Presumptive streptococcal colonies were sub-cultured onto blood agar plates (Becton Dickinson GmbH) to obtain pure isolates. Species-level identification was initially performed using MALDI-TOF mass spectrometry, followed by optochin susceptibility testing using a 15 µg optochin disc (Becton Dickinson GmbH). A zone of inhibition ≥14 mm was interpreted as optochin susceptible.

**S2. Molecular confirmation of *Streptococcus pneumoniae* by PCR**

All optochin-susceptible isolates underwent molecular confirmation by PCR targeting the *lytA* gene. DNA was extracted by suspending multiple bacterial colonies in 125 µL of sterile nuclease-free water with 5 µL of lysostaphin (2.6 mg/mL), followed by heat lysis at 96 °C for 10 min and centrifugation.

PCR primers targeting the 5′end of the *lytA* gene (25 nucleotides) were supplied by Conda Lab (Madrid, Spain); primer sequences are provided in Table 1 in the main manuscript. PCR reactions were performed in a final volume of 25 µL, containing 0.2 µL Taq polymerase (5 U/µL), 5 µL 5× Green GoTaq™ Flexi Buffer (Thermo Fisher Scientific), 2.5 µL MgCl₂ (15 mM), 2.5 µL dNTPs (2 mM), 1 µL of each primer (25 µM), 3 µL DNA template, and nuclease-free water.

Thermal cycling conditions consisted of an initial denaturation at 95 °C for 6 min, followed by annealing at 60 °C for 2 min and extension at 72 °C for 2 min. This was followed by 32 amplification cycles (94 °C for 75 s, 60 °C for 75 s, and 72 °C for 120 s), two final cycles (94 °C for 75 s and 60 °C for 75 s), and a final extension at 72 °C for 10 min.

PCR products were resolved by electrophoresis on a 1.8% agarose gel stained with SYBR Safe DNA Gel Stain (4 µL/100 mL) at 100 V, alongside a 100 bp DNA ladder (Invitrogen, Thermo Fisher Scientific). Bands were visualized using a Syngene G:Box blue-light or UV transilluminator.

**S3. Species differentiation within the *Streptococcus mitis* group**

Optochin-susceptible but *lytA*-negative isolates were further characterized using species-specific PCR assays targeting the *SPN001* (*Streptococcus pneumoniae*) and *SPPN_RS10375* (*Streptococcus pseudopneumoniae)* genes. Amplification of the *map* and/or *guaA* housekeeping genes was used as a group-level positive control for members of the *Streptococcus mitis* group. Primer sequences and PCR conditions are detailed in reference (1).

**S4. Azithromycin susceptibility testing**

Azithromycin susceptibility was determined using E-test strips (0.016-256 µg/mL; bioMérieux, Madrid, Spain). Bacterial suspensions adjusted to a 0.5 McFarland standard were inoculated onto Mueller-Hinton agar supplemented with 5% sheep blood (Becton Dickinson GmbH). Plates were incubated at 37ºC in 5% CO_2_ for 18-20 h. Minimal inhibitory concentration (MIC) values were interpreted according to Clinical and Laboratory Standards Institute (CLSI) guidelines (2). *S. pneumoniae* ATCC 49619 was used as a quality-control reference strain.

**S5. Isolate-based detection of macrolide resistance determinants**

PCR detection of macrolide resistance determinants was performed on *S. pneumoniae* isolates, including all azithromycin-resistant isolates (MIC ≥ 2 µg/mL) and a randomly selected subset of azithromycin-susceptible isolates (MIC < 2 µg/mL). Multiplex PCR targeted the *erm(B)* and *mef(A/E)* genes using previously described primers (Table 1).

Reference *S. pneumoniae* strains carrying *erm(B)* (strain 15604) and *mef(A/E)* (strain 15603), provided by Carmen Ardanuy (Department of Clinical Microbiology, Hospital Bellvitge, Barcelona, Spain), were used as positive controls. Genomic DNA from both reference strains was pooled in equimolar concentrations for use as a multiplex control. The genotypes of the reference strains were confirmed by singleplex PCR using established primers for *erm(B)* and *mef(A/E)* (3), designed as previously described (4).

Multiplex PCR reactions were prepared as described in Section S2, with optimized primer concentrations of 0.5 µM for *erm(B)* and 0.2 µM for *mef(A/E)*. Cycling conditions consisted of an initial denaturation at 93°C for 3 min, followed by 30 cycles of denaturation at 93°C for 1 min, annealing at 62°C for 1 min, and extension at 65°C for 4 min, with a final extension at 65°C for 3 min. Amplicons were visualized as described in Section S2.

**S6. DNA extraction for Oxford nanopore sequencing**

For isolates lacking detectable macrolide resistance genes by PCR, genomic DNA was extracted using a cetyltrimethylammonium bromide (CTAB) methodology described by Pinzauti *et al.* (5). Briefly, bacterial pellets were resuspended in TE buffer (10 mM Tris, 1 mM EDTA, pH 8.0) and incubated at 37 °C with lysozyme (2.6 mg/mL) for 60 min, followed by proteinase K (0.1 mg/mL) digestion in the presence of 0.5% sodium dodecyl sulfate (SDS) for 30 min.

DNA was purified by two chloroform/isoamyl alcohol extractions (24:1 v/v) and precipitated in 0.6 volumes of ice-cold isopropanol (-20ºC, 30 min). After drying and resuspension in 100 µL of nuclease-free water, DNA quality and concentration were assessed using a NanoDrop ND-1000 spectrophotometer (Thermo Fisher Scientific), and a Qubit 4 Fluorometer (Invitrogen), respectively.

The suspensions were then incubated at 65 °C for 10 min with 0.5 M NaCl and CTAB/NaCl solution (10% CTAB, 0.7 M NaCl) to precipitate proteins and polysaccharides. DNA was purified by two chloroform–isoamyl alcohol extractions (24:1, v/v) and precipitated with 0.6 volumes of ice-cold isopropanol (−20 °C, 30 min). After air-drying, DNA was resuspended in 100 µL of nuclease-free water. DNA concentration and purity were assessed using a Qubit 4 Fluorometer (Invitrogen) and a NanoDrop ND-1000 spectrophotometer (Thermo Fisher Scientific), respectively.

**S7. Oxford nanopore sequencing and bioinformatic analysis**

Whole-genome sequencing was performed on *S. pneumoniae* isolates using Oxford nanopore technology. Sequencing libraries were prepared with the Rapid Barcoding Kit (SQK-RBK114; Oxford Nanopore Technologies) and loaded onto a FLO-MIN114 flow cell on a MinION Mk1C platform (Oxford Nanopore Technologies). Raw POD5 files were basecalled into FASTQ format using Dorado (v0.9.1) with the high-accuracy model.

FASTQ files were processed using a custom Nextflow DSL2 pipeline (v25.04.6) (6) to generate de novo genome assemblies (assembly mode) (7). Long reads were quality-filtered with Filtlong (v0.3.1) (8) and trimmed using Porechop (v0.2.4) (9). Genome assembly was performed with Flye (v2.9.6) (10) followed by polishing rounds with Medaka (v2.1.1) (11). Assembled genomes were annotated for antimicrobial resistance genes using AMRFinder (12) and ResFinder (13).

**S8. Whole-sample characterization of the microbiome and resistome**

Of the 936 NPS samples collected in DNA/RNA Shield medium, 102 were selected for whole-sample sequencing based on their isolate-based *S. pneumoniae* carriage status and azithromycin MICs determined by E-test® (Figure 1) from the corresponding STGG-preserved NPS samples.

*DNA extraction*

DNA was extracted from each selected NPS sample using the KingFisher Flex automated platform with the MagMAX Microbiome DNA Isolation Kit (Life Technologies, CA, USA). DNA concentration and purity were assessed using a Quantus Fluorometer (Promega, Madison, WI, USA).

*Library preparation and sequencing*

Microbiome and resistome profiling were performed using a targeted next-generation sequencing approach with the Ion AmpliSeq™ Pan-Bacterial Research Panel (Thermo Fisher Scientific). The panel comprised two primer pools: Pool 1, including 269 amplicons targeting 21 bacterial species and 716 amplicons targeting 364 antimicrobial resistance genes (ARGs), and Pool 2, comprising 24 amplicons targeting variable regions of the 16S rRNA gene.

For library preparation, 10 ng of DNA was used for Pool 1 and 1 ng for Pool 2. Libraries were prepared using the Ion AmpliSeq Library Kit (Life Technologies, Carlsbad, CA, USA), pooled at equimolar concentrations, and loaded onto an Ion™ Chip using the Ion Chef Instrument. Template preparation and sequencing were performed on the Ion Torrent S5xl System (Thermo Fisher Scientific) according to the manufacturer’s protocols.

*Sequencing Data Analysis*

Demultiplexing was performed on the Ion Torrent Server software using unique sample barcodes. The PanBacterialAnalysis plugin in the Torrent Suite software was used for bacterial species identification, resistome profiling, and microbiome analyses under default parameters. For Pool 1, read counts mapping to ARGs and bacterial species were reported and annotated using the Comprehensive Antibiotic Resistance Database (CARD). Read counts for macrolide resistance genes were summed per sample. For Pool 2, reads were aligned to the Greengenes 16S rRNA reference database for microbiome profiling. All read counts were normalized to the total number of reads sequenced per sample.

**References**

1. **Basheer M, Sasic K, Carlsson A, Vestberg N, Henriques-Normark B, Blomqvist K, et al.** A novel LAMP-based assay for the identification of Streptococcus pneumoniae and Streptococcus pseudopneumoniae in clinical isolates. J Clin Microbiol. 2024;62(12):e00912-24.
2. **Clinical and Laboratory Standards Institute.** Methods for dilution antimicrobial susceptibility tests for bacteria that grow aerobically; approved standard—ninth edition. CLSI document M07-A9. Wayne (PA): CLSI; 2012.
3. **Sutcliffe J, Grebe T, Tait-Kamradt A, Wondrack L.** Detection of erythromycin-resistant determinants by PCR. Antimicrob Agents Chemother. 1996;40(11):2562-2566.
4. **Malhotra-Kumar S, Lammens C, Piessens J, Goossens H.** Multiplex PCR for simultaneous detection of macrolide and tetracycline resistance determinants in streptococci. Antimicrob Agents Chemother. 2005;49(11):4798-4800.
5. **Pinzauti D, Iannelli F, Pozzi G, Santoro F.** DNA isolation methods for Nanopore sequencing of the Streptococcus mitis genome. Microb Genom. 2022;8(2):000764.
6. **Di Tommaso P, Chatzou M, Floden EW, Barja PP, Palumbo E, Notredame C.** Nextflow enables reproducible computational workflows. Nat Biotechnol. 2017;35(4):316-319.
7. **AMRmicrobiology.** ONT_BACTERIAL_ANALYSIS: Nextflow pipeline for bacterial genome assembly and antimicrobial resistance profiling. 2023. Available from: <https://github.com/AMRmicrobiology/ONT_BACTERIAL_ANALYSIS>
8. **Wick RR.** Filtlong: quality filtering tool for long-read sequencing data. 2017. Available from: <https://github.com/rrwick/Filtlong>
9. **Wick RR.** Porechop: adapter trimming for Oxford Nanopore sequencing reads. 2017. Available from: <https://github.com/rrwick/Porechop>
10. **Kolmogorov M, Yuan J, Lin Y, Pevzner PA.**Assembly of long, error-prone reads using repeat graphs. Nat Biotechnol. 2019;37(5):540-546.
11. **Oxford Nanopore Technologies.** Medaka: sequence correction provided by ONT. 2025. Available from: <https://github.com/nanoporetech/medaka>
12. **Feldgarden M, Brover V, Gonzalez-Escalona N, Frye JG, Haendiges J, Haft DH, et al.**AMRFinderPlus and the Reference Gene Catalog facilitate examination of the genomic links among antimicrobial resistance, stress response, and virulence. Sci Rep. 2021;11:12728.
13. **Zankari E, Hasman H, Cosentino S, Vestergaard M, Rasmussen S, Lund O, et al.** Identification of acquired antimicrobial resistance genes. J Antimicrob Chemother. 2012;67(11):2640-2644.
